# Supplementary material for: Content-rich biological network constructed by mining PubMed abstracts
Source: BMC Bioinformatics. 2004 Oct 8;5:147. doi: 10.1186/1471-2105-5-147 (PMC528731; doi:10.1186/1471-2105-5-147)
Supplement: Additional File 5 — The original Chilibot query results of the term "long-term potentiation (LTP)" and 22 other terms, limiting the latest references analyzed to the years 1990, 1995, 2000, and 2004. [file 1471-2105-5-147-S5.bz2 › chilibotAdditionalFile5/ltp1995/html/PKA_ATF.html]

 


 **PKA** and **ATF** 
  
Found 5 abstracts in PubMed,  **5 abstracts were retrieved and analyzed**.  


---

 Search Google  |
 PDF files only 
|  EDU domain only 

---

**Interactive relationship** (e.g. stimulation, inhibition, etc)

- The CaMKIV mediated gene induction requires the activity of CREB  **ATF**  family members but is independent of  **PKA**  activity.  Ref: 8065343 Mol Cell Biol, 1994
- **ATF**  1 is as active as CREB in its ability to mediate the transcriptional effects of  **PKA** , and, because  **ATF**  1 has a smaller effect on basal expression, it is actually more responsive than CREB to cAMP.  Ref: 1655749 J Biol Chem, 1991
